# Altered brain activity and the effect of personality traits in excessive smartphone use during facial emotion processing

**DOI:** 10.1038/s41598-017-08824-y

**Published:** 2017-09-22

**Authors:** Ji-Won Chun, Jihye Choi, Jin-Young Kim, Hyun Cho, Kook-Jin Ahn, Jong-Ho Nam, Jung-Seok Choi, Dai-Jin Kim

**Affiliations:** 1 Department of Psychiatry, Seoul St. Mary’s Hospital, The Catholic University of Korea College of Medicine, Seoul, Republic of Korea; 2 Department of Radiology, Seoul St. Mary’s Hospital, The Catholic University of Korea College of Medicine, Seoul, Republic of Korea; 3 Department of Psychology, The Catholic University of Korea, Seoul, Republic of Korea; 4 Department of Psychiatry, SMG-SNU Boramae Center, Seoul, Republic of Korea

## Abstract

Excessive smartphone use is a phenomenon related to maladaptive smartphone use, leading to negative consequences. This study set out with the aim of assessing the effects of excessive smartphone use on behavioral and neural responses during facial emotional processing. We examined 25 excessive smartphone users and 27 normal control users using functional MRI during facial emotion processing and investigated Behavioral Inhibition System/Behavioral Activation System (BIS/BAS). The excessive smartphone use group (SP) showed neural deactivation in the dorsolateral prefrontal cortex (DLPFC) and dorsal anterior cingulate cortex (dACC) during the presentation of an angry face and emotional transition compared to that of the normal control group (NC). Additionally, the SP revealed neural deactivation of the superior temporal sulcus and temporo-parietal junction related to social interaction during emotional transition compared to the NC. We found that BAS-Reward Responsiveness level was correlated with behavioral responses during repeated happy faces related to emotional reward in SP compared to NC. It can thus be suggested that excessive smartphone use is likely to fail on cognitive control during emotional processing, and this impairment might be influenced on emotional processing related to social interaction.

## Introduction

Over the past decade, smartphones have become a necessity for people’s daily lives with the development of technology. Despite many positive aspects, excessive mobile phone use often leads to potentially harmful or disturbing behaviors such as uncontrolled use leading to a negative impact on various aspects of daily life^1^, and thus, problematic mobile phone use has raised sufficient concerns for being considered a potential public health issue^2^. People who are addicted to smartphone use are unable to maintain concentration on a task or in interpersonal relationships due to the need to constantly check mobile phone notifications^3^. Additionally, the tendency to be emotionally vulnerable^4–6^ and have low levels of self-esteem^4,7,8^ is known to be associated with increased smartphone addiction. In the study using neuroimaging, college students with mobile phone dependence showed altered gray matter volume and white matter integrity^9^. In the previous study using exploratory factor analysis, smartphone addiction symptoms were identified that disregarding of harmful consequences, preoccupation, inability to control craving, productivity loss, and feeling anxious and lost^10^. Particularly, lonely and depressed people preference interacting with other people by texting or social networking applications^10,11^ and these pattern leads to negative outcomes related to their Internet use^11^. Also, previous study using Internet questionnaire identified that lonely participants preferred making voice calls and anxious participants preferred texting^12^. Therefore, it would be suggested that individual’s emotion influence on excessive smartphone use associated with social interaction.

Although excessive smartphone use did not define clinical criteria for disorder, excessive smartphone use shares similar sub-dimension with addiction criteria of DSM 5^13,14^. In particular, the negative aspects of excessive smartphone use share the same effects as Internet addiction including Internet gaming addiction on interpersonal interaction^15^. In previous studies, Internet addiction and pathological Internet use have been shown to lead to negative outcomes including uncontrolled Internet use, tolerance, withdrawal, social isolation and poor academic or professional achievement^16,17^. However, the study of smartphone addiction as a potential psychiatric disorder is in its infancy, and the evidence supporting problematic smartphone use as an addictive behavior is still insufficient^1^.

Problematic smartphone use have related to individual’s social interaction. In previous studies, people using SNS more in terms of time spent usage were found to be less involved in the real life community^18^, and individuals who did not feel comfortable with their peers in real life tend to use social networking service (SNS) more in order to compensate^19^. In some studies, the negative consequences of using SNS use considered as criteria for substance dependence, and these might be considered as valid criteria for behavioral addiction^20^. Addiction influence on the brain’s neuronal circuits necessary for not only reward and motivation, but also social behaviors, and thus allows addicted individuals to make poor choices despite awareness of the negative outcomes^21^. Internet addiction criteria of DSM 5 have included social isolation, and previous studies related to Internet addiction, Internet gaming addiction, and smartphone addiction have considered important role of social interaction or interpersonal relationship on addiction^11,22,23^. It is necessary to take account of individual’s personality traits in order to understand characteristic of social interaction in smartphone addiction. In particular, personality traits related to emotional processing are key to understanding social interaction.

It is known that the behavioral inhibition (BIS) and behavioral activation (BAS) systems are closely related not only to temperament and personality traits but also to a wide range of affective experiences^24^. The BAS are associated with positive emotional and extroversion, whereas BIS are closely related to negative emotional and emotional instability^25^. Furthermore, BAS and BIS are explained on the basis of the independent and distinctive structures in the nervous system and behavioral patterns^26^. According to Gray’s theory, the BIS/BAS systems are theoretical biopsychological systems related to personality traits involving sensitivity toward stimuli associated with negative and positive reinforcement and regulation of motivational behavior^27–29^. In particular, it is reported that BIS is sensitive to punishment and non-reward cues in terminating behavioral output, and BAS is not only sensitive to reward cues and activating goal directed behavior^28,30,31^, but also likely to promote the experience of positive feelings such as exaltation and happiness^24^. Additionally, BAS are considered to be personality factors associated with Internet addiction^32,33^ and smartphone dependency^14,34^. In previous studies, BAS activity is associated with substances use such as alcohol^35–37^. It has been reported that BIS and BAS were associated with the neural activity in the lateral prefrontal cortex^38^ and dorsal anterior cingulate cortex (dACC)^39^ related to cognitive control. Therefore, it can be supposed that individual personality traits in excessive smartphone use have an influence on behavioral and neural response for social reward cue.

In this study, we have designed a task to explore altered brain activity in excessive smartphone use during cognitive control of an emotional face. In previous studies, a facial emotion discrimination task has been generally used to study social interaction^40,41^. The perception of changes due to facial movements plays a more central role in social communication^41^. In previous studies, an increased level of general anxiety, including social anxiety, is related to excessive smartphone use^8,42,43^, and socially anxious individuals revealed attentional biases toward threatening stimuli, especially angry faces^44^. In previous neuroimaging studies, the dorsolateral prefrontal cortex (DLPFC) and dACC have been shown to be engaged in cognitive control and emotional regulation^45–49^. Previous studies using animal models of affective learning and imaging studies of either cognitive control or emotional responding in both healthy and psychiatric populations have implicated regions of the prefrontal cortex (PFC) and anterior cingulate cortex (ACC)^50–53^. The DLPFC may be more involved in maintaining a representation of the context, such as goals, rules and sequence of events, necessary to perform a task accurately^54,55^. Additionally, the ACC has been shown to be activated by the manipulation of interference and cognitive loading in a previous study related to working memory^54^. Activation of this region is thought to be related to detecting cognitive conflict and signaling the need for greater allocation of attention for the purpose of resolving conflict^56–58^.

In this study, we will focus on identifying altered brain activity involved in social interaction in those with excessive smartphone use. Although the relationship between excessive smartphone use and social interaction has been reported^19,20^, no evidence has been found proving altered neural activity of social emotion in excessive smartphone use. Therefore, we investigated the differences in the behavioral and neural responses between the excessive smartphone use group (SP) and the normal control group (NC) in the cognitive control of facial expressions in order to find neurobiological evidence of excessive smartphone use affecting social interaction. Additionally, this study tends to investigate the influence of personality trait related reward system on the emotional processing due to social context in excessive smartphone use. We examined the correlations between BAS reward response and the behavioral and neural responses related to facial emotion processing in the SP compared with NC.

This study investigated the effect of excessive smartphone use on neural activity during facial emotion discrimination through the following hypothesis. First, we hypothesized that the SP would show a cognitive deficit during the emotional transition of faces requiring fast emotional regulation. Second, we expected that there is altered neural activity in the SP compared to that in the NC in the prefrontal and cingulate cortex related to emotional regulation and cognitive control. Lastly, we hypothesized that BAS-Reward Responsiveness (BAS-RR) is correlated with the responses of happy faces related to the emotional reward in the SP.

## Results

### Demographics and clinical data

Table 1 summarizes the demographic and clinical characteristics of the two groups. The two groups did not differ in age, K-WAIS, and the main usage of smartphones, whereas the time of smartphone use per week, *t*(50) = 4.67, *p* < 0.001, the time of major smartphone use per week, *t*(50) = 3.47, *p* < 0.005, and Smartphone Addiction Proneness Scale (SAPS) scores, *t*(50) = 4.55, *p* < 0.001 were significantly different. The SP showed higher score on the BIS, *t*(50) = 3.6, *p* < 0.001 and BAS, *t*(50) = 5.38, *p* < 0.001, and particularly the SP revealed higher score on the BAS-RR, *t*(50) = 2.32, *p* < 0.005 and BAS-Fun Seeking (BAS-FS), *t*(50) = 5.08, *p* < 0.001 compared to NC. Additionally, there was a significant difference in the education duration, *t*(51) = 2.16, *p* < 0.05, but the difference was around a year. According to gender distribution, there was no significant difference across groups.

**Table 1:** Demographic characteristics of the PSU and NC.

	SP (n = 25)		NC (n = 27)		t score
	mean	SD	mean	SD	
Age	27.76	5.97	28.93	6.39	−0.61
K-WAIS	111.84	10.59	113.04	10.53	−0.41
Education duration (years)	15.12	1.42	15.93	1.27	−2.16*
Gender					
Male	52.00%	(n = 13)	66.70%	(n = 18)	x² = 1.160
Female	48.00%	(n = 12)	33.30%	(n = 9)	
Duration of smartphone use (year)	4.60	1.29	4.85	1.63	−0.61
Time for smartphone use per week (hours)	44.96	26.05	15.7	18.84	4.67***
Time for major smartphone use per week (hours)	24.79	21.15	9.8	7.17	3.47**
Major usage of smartphone					
Internet searching	12.00%		25.90%		x² = 5.063
Social network service	64.00%		55.60%		
Entertainment	12.00%		18.50%		
etc.	12.00%		0%		
SAPS	41.72	4.77	20.41	4.55	16.48***
BIS scale	20.52	3.04	17	3.6	3.8***
BAS scale	36.76	4.02	31.59	5.38	3.9***
Reward responsiveness	14.96	2.34	12.93	2.32	3.15**
Drive	10.48	1.5	10.11	2.55	0.63
Fun seeking	11.32	1.65	8.56	2.21	5.08***
Economic status					
Upper	12.00%		22.20%		x² = 2.408
Upper-middle	12.00%		11.10%		
Middle	28.00%		37.00%		
Lower-middle	32.00%		22.20%		
Lower	16.00%		7.40%		

Abbreviations: SP, Excessive smartphone use group; NC, Normal control group; SAPS, Smartphone Addiction Proneness Scale; BIS, Behavioral inhibition system; BAS, Behavioral activation system.

*p < 0.05, **p < 0.005, ***p < 0.001.

### Behavioral performance

**Figure 1:**
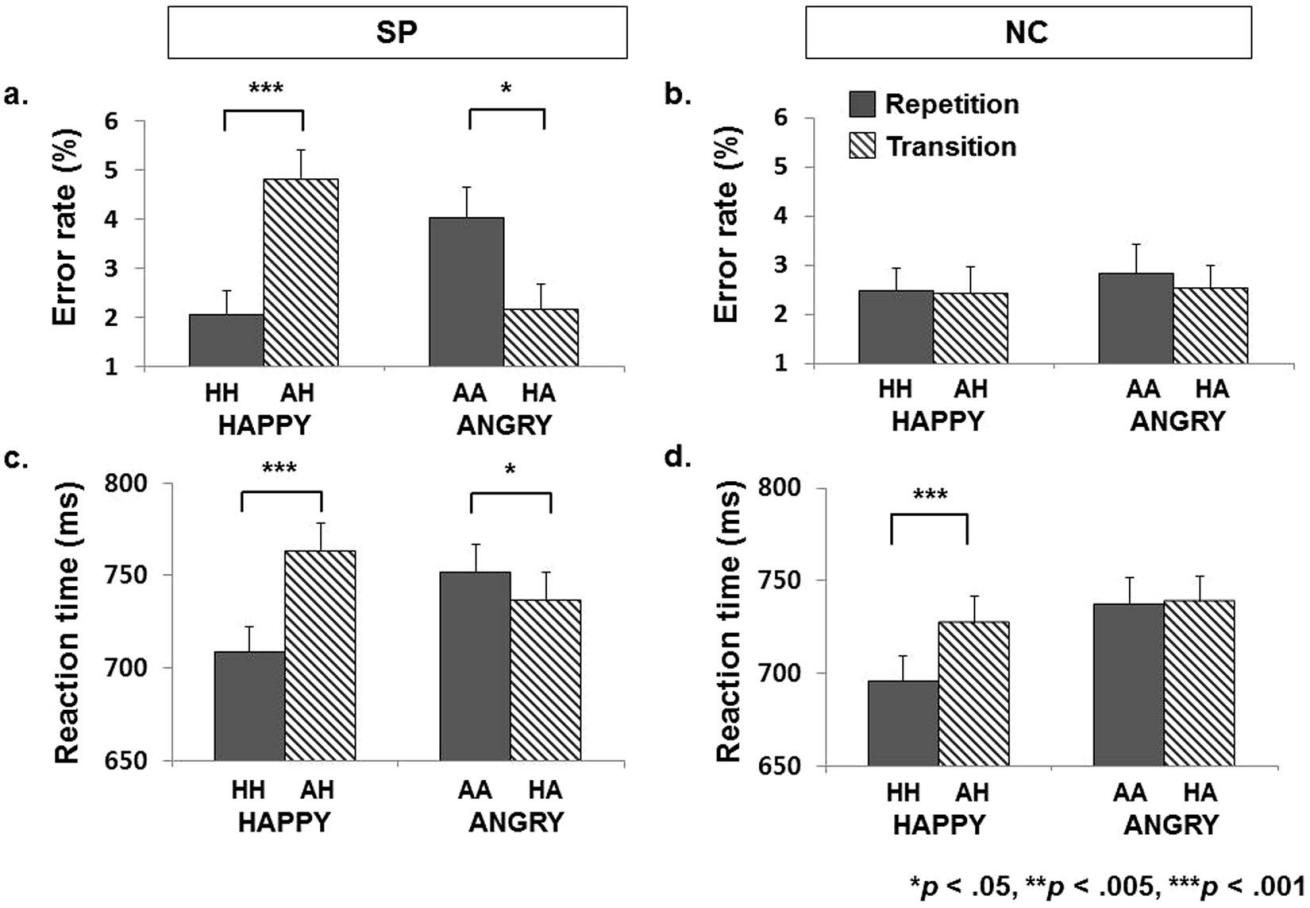
Behavioral responses of each group. In the SP, the error rate for AH was higher than that for the HH, and the error rate for the AA was higher than that for the HA (**a**); however, there were no significant differences between conditions in the NC (**b**). The SP showed a higher error rate than the NC in the AH. The SP exhibited slower responses in the AH than in the HH, and they also showed slower responses in the AA than the HA (**c**). The NC showed delayed responses in the AH trials compared to that in the HH trials, however, there were no significant differences between the AA and the HA in the NC (**d**).

As shown in Table 2 and Fig. 1, we conducted a repeated-measures ANOVA on the error rate with emotional valence of face (happy and angry), emotional status (repetition vs. transition), and group (SP and NC). For error rate, there was a 3-way interaction between the emotional valence, emotional status, and groups, *F*(1,50) = 11.52, *η* ^2^ = 0.91, *p* < 0.001. The ANOVA revealed significant interaction between the emotional valence and emotional status of the face, *F*(1,50) = 14.19, *η* ^2^ = 0.96, *p* < 0.001.

**Table 2:** Behavioral responses.

			SP (n = 27)		NC (n = 25)		t score
			mean	SD	mean	SD	
**Error rate (%)**							
Happy	repetition	HH	2.07	2.05	2.5	2.58	−0.662
	transition	AH	4.83	3.5	2.43	2.06	3.045*
Angry	repetition	AA	4.02	3.92	2.83	2.33	1.34
	transition	HA	2.17	2.51	2.52	2.5	−0.501
**Reaction time (ms)**							
Happy	repetition	HH	708.61	71.23	695.91	67.05	−0.662
	transition	AH	763.7	73.59	727.68	73.36	1.767
Angry	repetition	AA	752.01	76.59	737.16	72.52	0.718
	transition	HA	737.57	68.77	739.12	70.21	−0.08

Abbreviations: SP, Excessive smartphone use group; NC, Normal control group; HH, Happy face followed by happy face; AH, Angry face followed by happy face; AA, Angry face followed by angry face; HA, Happy face followed by angry face.

In the SP, the error rate for the AH was higher than that for the HH, *t*(24) = 4.67, *p* < 0.001, and the error rate for the AA was higher than that for the HA, *t*(24) = 2.50, *p* < 0.05; however, there were no significant differences between conditions in the NC. In the group comparison, the SP showed a higher error rate than the NC in AH, *t*(50) = 2.04, *p* < 0.005.

For the reaction time, there were main effects for the emotional valence of the face, *F*(1,50) = 20.05, *η* ^2^ = 0.99, *p* < 0.001, and emotional status, *F*(1,50) = 33.32, *η* ^2^ = 1.00, *p* < 0.001. There were a 3-way interaction among the two conditions and the groups, *F*(1,50) = 13.03, *η* ^2^ = 0.94, *p* < 0.005. There was a significant interaction in the reaction time between the emotional valence of the face and the groups, *F*(1,50) = 5.14, *η* ^2^ = 0.60, *p* < 0.05. There was a significant interaction between the emotional valence and emotional transition of the face, *F*(1,50) = 81.46, *η* ^2^ = 1.00, *p* < 0.001.

The SP exhibited slower responses in the AH than in the HH, *t*(24) = 8.45, *p* < 0.001, and they also showed slower responses in the AA than the HA, *t*(24) = 2.77, *p* < 0.05. The NC showed delayed responses in the AH trials compared to that in the HH trials, *t*(26) = 5.96, *p* < 0.001, however, there were no significant differences between the AA and HA in the NC.

**Figure 2:**
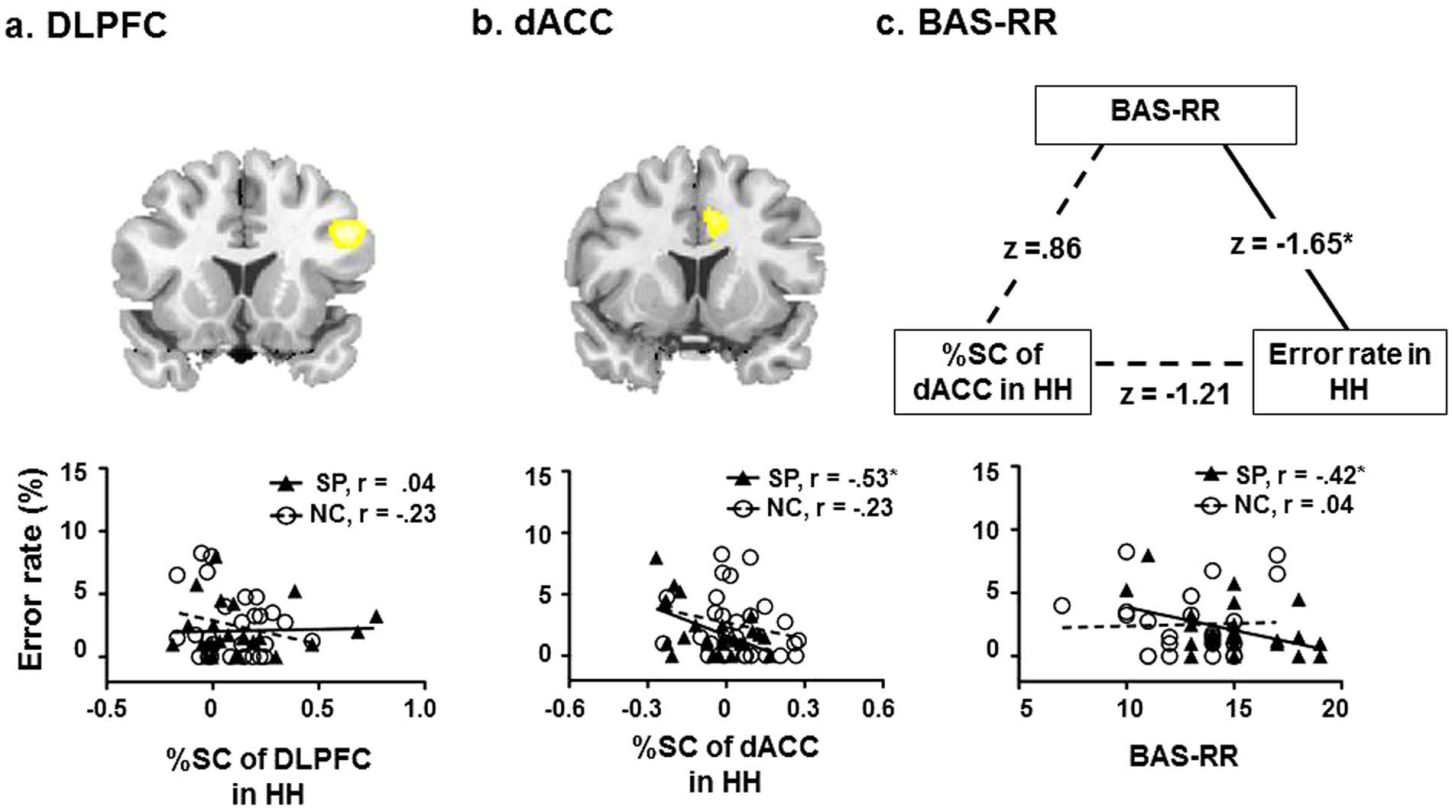
Correlations among neural activity of the ROIs, the error rate, and the BAS-RR scores. In the SP, the activation of the right DLPFC did not showed correlation with behavioral error (**a**), and the dACC exhibited negative correlation with the error rate under HH, *r* = −0.53, *p* < 0.05 in SP (**b**). In the SP compared to NC, the error rate revealed a significant negative correlation with BAS-RR score during the HH, *r* = −0.42, *p* < 0.05 (**c**).

Regarding to correlation between the behavior response and effect of personality trait, BAS-RR score revealed a significant negative correlation with the error rate during the HH, *r* = −0.42, *p* < 0.05, in SP, however there was no significant correlation in NC (Fig. 2). The results were converted to equally probable z-scores, comparing Fisher’s z-transformed correlation values across groups (z = 1.65, *p* = 0.049).

### Functional MRI results

#### Group differences in emotional valence

The results from the emotional valence of the face condition analysis are presented in Table 3. In the happy face condition, the SP showed less activity in the left precentral gyrus (PG), left lingual gyrus (LG), right inferior temporal gyrus, left middle frontal gyrus (MFG), and left middle temporal gyrus than the NC. In the angry face condition, the SP exhibited less activity in the bilateral precentral gyri, left LG, bilateral middle temporal gyri, right superior parietal gyrus (SPG), left middle occipital gyrus, right dACC, left DLPFC, right supplementary motor area (SMA), right cuneus, right thalamus, left cerebellum, left superior occipital gyrus (SOG), and left MFG than the NC. However, the SP did not exhibit significantly more activity than the NC in either the happy or angry face condition.

**Table 3:** Group differences of brain regions showing significant activation in each emotional valence.

Region	Coordinates			T-score	No. of Voxels
	x	y	z		
**HAPPY face**					
*SP* > *NC*					
None					
*SP* < *NC*					
L. Precentral gyrus	−52	2	40	10.12	342
L. Lingual gyrus	−14	−80	−2	8.93	319
R. Inferior temporal gyrus	48	−72	−4	7.58	122
L. Middle frontal gyrus	−42	40	24	7.58	171
L. Middle temporal gyrus	−36	−54	62	7.36	217
**ANGRY face**					
*SP* > *NC*					
None					
*SP* < *NC*					
B. Precentral gyrus	−52	4	46	10.03	366
	48	6	52	9.35	301
L. Lingual gyrus	−18	−80	−4	9.47	761
B. Middle temporal gyrus	42	−68	−2	9.03	205
	−56	−52	10	8.66	807
R. Superior parietal gyrus	38	−52	62	8.74	694
L. Middle occipital gyrus	−38	−88	−2	9.2	365
R. dorsal ACC	4	12	32	8.58	338
L. DLPFC	−42	38	20	8.43	190
R. SMA	2	−4	66	7.63	402
R. Cuneus	6	−88	14	7.84	210
R. Thalamus	18	−10	6	7.73	372
L. Cerebellum	−20	−60	−14	7.67	118
L. Superior occipital gyrus	−24	−90	30	7.46	271
L. Middle frontal gyrus	−24	50	34	7.4	239

Clusters with peak-level and FWE-corrected p < 0.001 and more than 100 voxels are reported.

Abbreviations: L., Left; R., Right; B., Bilateral; ACC, Anterior cingulate cortex; DLPFC, Dorsolateral prefrontal cortex; SMA, Supplementary motor area; FWE, family wise error.

#### Group differences in emotional status

The results from the analysis of the emotional status are presented in Table 4. The SP showed less activity in the right inferior temporal gyrus, left LG, and left PG than the NC with emotional repetition. The SP exhibited less activity in the bilateral precentral gyri, left LG, left STS, left SPG, left DLPFC, right interior temporal gyrus, left SMA, left inferior frontal gyrus, left supramarginal gyrus, left dorsal ACC, right TPJ, left inferior parietal gyrus, left SOG, right cuneus, and right superior frontal gyrus (SFG) with emotional transition. However, the SP did not exhibit significantly more activity than the NC with either emotional repetition or transition.

**Table 4:** Group differences of brain regions showing significant activation in emotional status.

Region	Coordinates			T-score	No. of Voxels
	x	y	z		
**Emotional repetition**					
*SP* > *NC*					
None					
*SP* < *NC*					
R. Inferior temporal gyrus	46	−72	−4	8.94	167
L. Lingual gyrus	−16	−80	−2	8.61	374
L. Precentral gyrus	−52	4	46	8.21	147
**Emotional transition**					
*SP* > *NC*					
None					
*SP* < *NC*					
B. Precentral gyrus	−50	2	42	12.98	811
	48	8	50	9.36	257
L. Lingual gyrus	−16	−82	−4	10.62	698
L. Superior temporal sulcus	−50	−52	6	9.68	2108
L. Superior parietal gyrus	−30	−66	60	9.34	270
L. DLPFC	−44	40	22	9.44	749
R. Inferior temporal gyrus	42	−66	−2	8.97	149
L. Supplementary motor area	−6	0	68	8.47	482
L. Inferior frontal gyrus	−44	24	2	8.19	137
L. Supramarginal gyrus	−54	−50	30	8.6	214
L. dorsal ACC	0	14	32	7.89	380
R. Tempro-parietal junction	62	−56	20	7.84	173
L. Inferior parietal gyrus	−44	−46	56	7.46	566
L. Superior occipital gyrus	−24	−90	30	7.24	109
R. Cuneus	4	−90	16	6.89	101
R. Superior frontal gyrus	4	34	46	6.65	101

Clusters with peak-level and FWE-corrected p < 0.001 and more than 100 voxels are reported.

Abbreviations: L., Left; R., Right; B., Bilateral; STS, Superior temporal sulcus; ACC, Anterior cingulate cortex; DLPFC, Dorsolateral prefrontal cortex; SMA, Supplementary motor area; TPJ, Tempro-parietal junction; FWE, family wise error.

#### Regional differences between conditions within groups

The results from the analysis of the reginal differences between conditions for each group are presented in Table 5. The SP showed stronger activation in the right SFG, right LG, left middle occipital gyrus, right dorsal ACC, right MFG, right thalamus, right postcentral gyrus, right superior temporal gyrus, and left inferior parietal gyrus during AH than during HH. Additionally, they revealed more activation of the right SFG, right DLPFC, left SPG, right inferior parietal gyrus, and right angular gyrus during AA than during HA. There were no significant differences in activity dependent on the condition in the SP. In contrast, the NC showed stronger activation in the left SPG, left SMA, left LG, and right SOG during AH than during HH. There were no significant differences dependent on other conditions in the NC.

**Table 5:** Brain regions of each group showing significant activation in conditions.

Region	Coordinates			T-score	No. of Voxels	Condition
	x	y	z			
***SP***						
R. Superior frontal gyrus	28	−4	62	6.85	458	HH < AH
R. Lingual gyrus	6	−90	2	6.33	659	HH < AH
L. Middle occipital gyrus	−30	−68	32	5.82	72	HH < AH
R. dorsal ACC*	6	10	38	5.8	136	HH < AH
R. Middle frontal gyrus	34	48	22	5.71	141	HH < AH
R. Thalamus	14	−20	12	5.56	77	HH < AH
R. Postcentral gyrus	30	−42	66	5.42	503	HH < AH
R. Superior temporal gyrus	46	−22	12	5.42	148	HH < AH
L. Inferior parietal gyrus	−34	−52	52	5.36	102	HH < AH
R. Superior frontal gyrus	12	36	38	6.88	490	AA > HA
R. DLPFC*	48	16	32	6.47	333	AA > HA
L. Superior parietal gyrus	−38	−68	54	6.01	291	AA > HA
R. Inferior parietal gyrus	50	−50	36	5.79	190	AA > HA
R. Angular gyrus	38	−74	42	5.77	251	AA > HA
***NC***						
L. Superior parietal gyrus	−28	−60	44	6.31	517	HH < AH
L. SMA	−4	0	66	6.23	181	HH < AH
L. Lingual gyrus	−8	−90	−6	6.2	400	HH < AH
R. Superior occipital gyrus	18	−96	4	5.37	76	HH < AH

Clusters with peak-level and FWE-corrected p < 0.05 and more than 100 voxels are reported.

Abbreviations: L., Left; R., Right; B., Bilateral; ACC, Anterior cingulate cortex; DLPFC, Dorsolateral prefrontal cortex; SMA, Supplementary motor area; FWE, family wise error; *Region of Interest.

#### ROIs analysis

**Figure 3:**
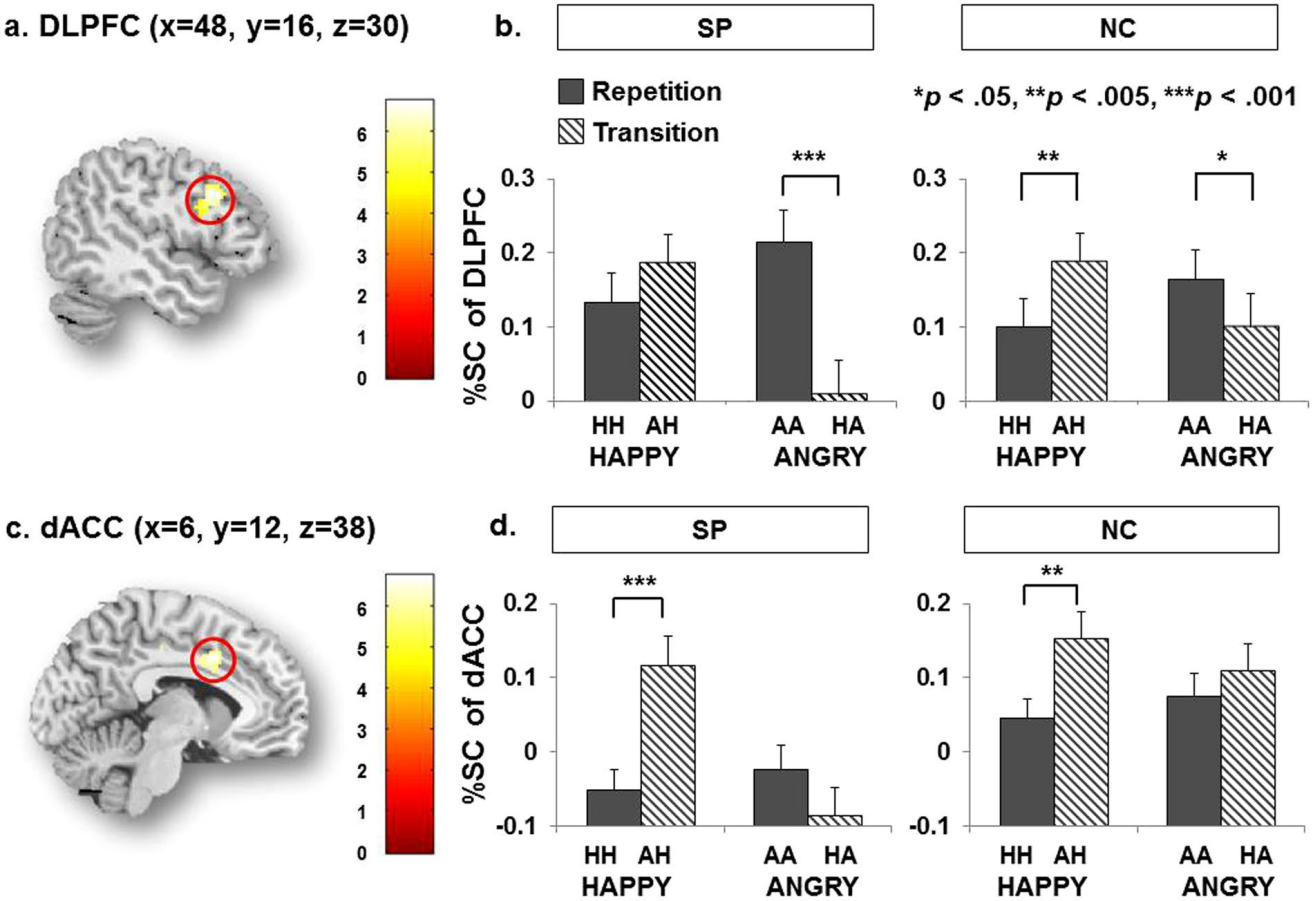
Neural activity in the DLPFC and dACC of each group. The right DLPFC extracted from the contrast between HH and AH in SP (**a**), and the right DLPFC in the SP showed stronger activity with emotional repetition than emotional transition, while the difference was not significant in the NC. Additionally, the NC showed greater activity in AH than in HH, while the difference was not significant in the SP (**b**). The right dACC extracted from the contrast between AA and HA in SP (**c**). In the right dACC, the SP showed greater activity in the happy than the angry faces, but the NC did not show a significant difference. Additionally, the SP revealed deactivation of the dACC in the HH, AA, and HA trials compared to observed in the NC (**d**).

In the region of interest (ROI) analysis for exploring the activation differences of the DLPFC (Fig. 3), the main effect of the emotional status of the face was significant, *F*(1,50) = 4.75, *η* ^2^ = 0.57, *p* < 0.05, and the activation of DLPFC during emotional repetition was higher than that during emotional transition. The interaction between the emotional status and group was significant, *F*(1,50) = 8.90, *η* ^2^ = 0.83, *p* < 0.005. Also, the interaction between the emotional valence of the face and the emotional status was significant, *F*(1,50) = 48.35, *η* ^2^ = 1.00, *p* < 0.001.

In the multiple comparison, the right DLPFC in the SP showed stronger activity with emotional repetition than emotional transition, *t*(24) = 3.22, *p* < 0.05, while the difference was not significant in the NC. In the AH, the NC showed greater activity in the right DLPFC than in HH, *t*(26) = 3.90, *p* < 0.005, but the SP did not show a significant difference in activity between the AH and HH.

In terms of the activity in the right dACC (Fig. 3), the main effects of emotional valence of the face, *F*(1,50) = 8.37, *η* ^2^ = 0.81, *p* < 0.005, and emotional status, *F*(1,50) = 16.58, *η* ^2^ = 0.98, *p* < 0.001, were significant. The activation of dACC during happy face trials was higher than that during angry face trials, and the activation of dACC during emotional transition was higher than that during emotional repetition. The 3-way interaction among the emotional valence, emotional status, and the groups was significant, *F*(1,50) = 6.43, *η* ^2^ = 0.70, *p* < 0.05. The interaction between the emotional valence of the face and the group was significant, *F*(1,50) = 6.15, *η* ^2^ = 0.11, *p* < 0.05. Additionally, the interaction between the emotional valence and emotional status of the face was significant, *F*(1,50) = 23.51, *η* ^2^ = 1.00, *p* < 0.001. In the right dACC, the SP showed greater activity in the happy than the angry faces, *t*(24) = 3.23, *p* < 0.001, but the NC did not show a significant difference.

In the SP, multiple comparisons revealed that the activation of the right dACC exhibited negative correlation with the error rate under HH, *r* = −0.53, *p* < 0.05 (Fig. 2), however, right DLPFC did not show a significant correlation between the BAS score and regional activations.

## Discussion

The aim of this study was to identify the behavioral differences between the SP and NC, and the altered brain activation of the prefrontal and cingulate cortex associated with cognitive control in SP compared to that of NC during facial emotion processing. Additionally, we identified the correlations between the BAS-RR activity, behavioral, and neural response during facial emotion processing related to emotional reward. We hypothesized that, based on the vulnerability of the excessive smartphone user in social interaction, SP would reveal deficit of cognitive control in behavioral and neural responses during facial emotional processing. In addition, we predicted that reward sensitivity in the SP would influence on cognitive processing of social reward cue.

In this study, the SP showed higher error rate induced by failure of cognitive control during presentations of angry face in the previous trial, whereas the NC did not show such a difference. These results indicated that the SP suffered difficulty on cognitive control caused by emotional evaluation when they are exposed to negative emotional expression. In particular, the SP showed a higher error rate under the emotional transition preceded by a negative emotional face, compared to the NC. These behavioral findings would indicate that the previous exposure to a negative emotion influenced the cognitive control on the current emotional transition trial in the SP.

In the neural response, the SP showed decreased activation in the dACC and DLPFC related to cognitive control of facial emotion compared to NC under angry face and emotional transition. These results associated with previous studies which reported dysfunction of frontolimbic region related to cognitive control in Internet gaming disorder^59^. The previous studies have reported that cognitive reappraisal of negative emotion activates the dACC and PFC systems that support the selection and application of reappraisal strategies and modulate activity in appraisal systems suitable for the goal of reappraisal^53,60–65^. In the results of meta-analysis, it has been reported that emotional interference during cognitive conflict induced neural activities in the DLPFC and dACC^66^. Therefore, decreased activity of both the dACC and DLPFC in the SP suggests that the cognitive control of negative emotion and emotional transition has been altered compared to that in the NC. Consistent with neural activities, the SP reported higher error in emotional transition after angry face comparted to NC. Therefore, excessive smartphone user has a harmful effect on cognitive control during emotional face processing, and this impairment might be influenced on emotional processing related to social interaction. Additionally, the SP revealed neural deactivation of the STS and TPJ compared to the NC. According to a previous study related to facial information, the activation of the STS is associated with processing and reacting to the emotional state of another person^41^. Additionally, it has been known that the right TPJ is selectively recruited for the attribution of mental states when receiving socially relevant stimuli^67,68^ and is more responsive to mentalizing than physical judgments^69^. Therefore, this evidence suggests that activation of the STS and TPJ during emotional transition reflects the social cognitive effort to make a rapid emotional judgment, and the SP showed a lower neural response towards emotional information involved in social context than the NC.

In the ROIs comparison, we found that NC showed higher activation of DLPFC during presentations of angry face in previous trial than during presentations of happy face in the previous trial, while the SP did not show significant difference of neural activation between the previous emotional valences under current happy face. Cognitive reappraisal has been known to enhance the signal in the DLPFC regions in cognitive regulation of negative emotion^70^. In a previous study on emotional distracters, normal control participants were able to recruit the DLPFC; however, depressed individuals showed an exaggerated amygdala response to such distracters and a failure to recruit the DLPFC^71^. In this study, the activation of DLPFC during emotional repetition was higher than that during emotional transition. The effect of repeated emotion revealed only in the angry face, not in the happy face. In particular, SP showed strong activation in DLPFC during repeated angry face compared to non-repeated angry face. In the previous study related to emotional processing, the repeated negative stimuli induced significant activation in the DLPFC, and functional connectivity between the DLPFC and other regions^72^. The activation of DLPFC can be regarded as a cognitive effort in other to process repeated angry faces. In previous studies, the right lateral prefrontal regions have been shown to be activated under interference conditions^73,74^, and the activation of right DLPFC region negatively correlated with sensitivity of interference^54^. Despite the higher DLPFC activation, the SP showed more behavioral errors during the repeated angry face than non-repeated angry face. Therefore, it would imply that the neural activity related emotional regulation fails to control the behavioral performance in excessive smartphone user.

The SP also showed less activation of the dACC, which is related to conflict monitoring, during presentations of angry faces than the NC and compared to happy faces within the SP. The dACC has been known to be associated with detecting cognitive conflict^47,57,75,76^ and signaling the need for greater allocation of attention for the purpose of resolving conflict^54^. In the previous study related to altered brain structure of mobile phone dependence (MPD), MPD individuals had decreased gray matter volume relative to controls, and they showed decreased white matter integrity of bilateral hippocampal cingulum bundle fibers^9^. Therefore, it can be inferred that the SP have a deficit in cognitive monitoring during the presence of negative emotional faces compared to NC and, as this result, the SP revealed higher error rate under angry face followed by happy face compared to NC. Additionally, the SP showed a higher error with less neural activity in the dACC related to cognitive monitoring during repeated happy face, while there was no significant correlation between error rate and activity in the DLPFC related to cognitive conflict resolution. In other words, it imply that the SP showed individual differences according to cognitive monitoring during repeated happy face related to emotional reward.

In this study, correlations between the BAS-RR level, behavioral and neural response of facial emotion were shown to depend on the group. In the correlation results, high BAS-RR individuals in the SP exhibited low error rate during repeated happy face. In particular, the correlation between the BAS-RR and error rate in repeated happy face showed a significant difference between the SP and NC. These results indicate that sensitivity of reward more influence behavioral performance during repeated positive facial expression in the SP compared to NC. Therefore, it can be supposed that high BAS-RR individuals in the SP are sensitive to emotional reward such as happy face, and they might use social network service in order to gain positive responses. This is related to a previous finding that people who have a negative social identity tend to use SNSs more in order to compensate for this^19^.

Finally, a number of important limitations need to be considered. First, the participants did not evaluate the emotional valence and arousal of each face in this study. Second, the main usages of smartphone were heterogeneous among the participants. A future study with more focus on problematic social network service use through smartphones is therefore suggested. Third, the gender differences in excessive smartphone use group were not considered. In subsequent study, it is necessary to identify differences in facial emotional discrimination according to gender differences in the SP using the same gender distribution. Additionally, we suggest that the functional connectivity of fronto-cingulate regions in SP are investigated in social cognitive contexts in future studies.

In summary, we showed that the SP exhibited different behavioral responses and functional alterations compared to the NC during emotional processing of faces. The SP revealed a cognitive deficit during the emotional transition preceded by a negative emotional face, compared to NC. In the neural activity, the SP showed a neural deactivation of prefrontal and cingulate cortex related to conflict detection and cognitive control compared to that of the NC during exposure to angry faces and emotional transition. The behavioral performance in the SP correlated with the activity of dACC related to cognitive monitoring during repeated happy face associated with emotional reward. Lastly, we found BAS-RR level was correlated with behavioral responses during repeated happy faces related to emotional reward in SP compared to NC. These findings may help us to understand altered neural responses associated with cognitive control during facial emotional processing, and could provide important implications for the effect of personality traits related to emotional reward in excessive smartphone use.

## Methods

### Participants

This study was conducted for adult men and women aged 19–35 through online recruiting. A total of 728 adults participated in the online survey on smartphone usage. Twenty-six adults with SP (14 male and 12 female) and 30 NC (18 male and 12 female) were recruited for the fMRI study, and all participants underwent the Mini-International Neuropsychiatric Interview by a clinician to screen out participants with a current psychiatric diagnosis. One participant was excluded because of depressive disorder, and the data from three participants were excluded because of severe head motion during the analysis; thus, the data from twenty-five adults with problematic smartphone use (13 male and 12 female, 27.76 ± 5.97 years) and twenty-seven NC (18 male and 9 female, 28.93 ± 6.39 years) were considered in this study (Table 1). Exclusion criteria included past or current major medical disorders (e.g., diabetes mellitus), neurological disorders (e.g., seizure disorders, head injury) or psychiatric disorders (e.g., major depressive disorder, anxiety disorders). All participants had normal or corrected-to-normal vision and were right-handed assessed by the Edinburgh handedness inventory^77^. The purpose and procedure of this study were explained to the participants. Each participant provided written informed consent, and this study was approved by the Institutional Review Board of Seoul St. Mary’s Hospital. All experiments were performed in accordance with relevant guidelines and regulations.

### Questionnaires

#### SAPS

Excessive smartphone use was estimated using SAPS developed by the Korean National Information Society Agency in 2011 and the reliability test of the scale yielded a Cronbach’s alpha of 0.814^78^. The SAPS is a self-report scale and includes fifteen items, and the responses are scored on a four-point Likert scale (1: Not at all to 4: Always). The SAPS has four subscales: disturbance of adaptive functions, virtual life orientation, withdrawal, and tolerance, and participants were classified as SP if their total score exceeded 44, or if their subscales scores exceeded 15, 13, and 13 for disturbance of adaptive function, withdrawal, and tolerance, respectively.

#### BIS/BAS

BIS and BAS are general motivation systems that underlie behavior and affect^28^. The BIS responds to cues associated with punishment; the BAS responds to those associated with reward. The BIS and BAS questionnaire scales assess BIS (7 items) and three subdomains of BAS-D (4 items), BAS-FS(4 items), and BAS-RR (5 items)^31^. Responses used a 4-point scale (1: strongly disagree, 4: strongly agree). Items on the BIS scale assess sensitivity to the mechanism controlling aversive motivation. Items in the BAS-RR subscale assess positive responses to anticipated rewards. Items in the BAS-D subscale assess persistent pursuit of desired appetitive goals. Items in the BAS-FS subscale assess desire for new rewards and willingness to spontaneously approach potentially rewarding events^14^.

### Facial emotion discrimination task

Participants performed a facial emotion discrimination task using Korean emotional faces, which were selected from the Korean Facial Expressions of Emotion^79^. To maintain the participants’ attention, the stimulus material for each trial consisted of a positive or negative emotional face on the left or right side and a fixation cross at the center of the gray background. Half of the participants were asked to press a button with their left or right index finger in response to a positive or negative feeling produced by the picture, respectively, regardless of the picture’s location. The other half of the participants were assigned to respond in the opposite manner as a counterbalance. The trials consisted of four different stimuli according to emotional valence (happy vs. angry) and emotional status (repetition vs. transition) of face. The task sequence was separated into two sessions and was composed of a rapid event-related design in which the duration of each trial was 1,500 ms and the inter-trial intervals were varied from 500 to 4,500 ms. Each session started with a 12-s dummy scan with six practice trials and included 160 events consisting of 40 repeated happy face trials, 40 non-repeated happy face trials, 40 repeated angry face trials, and 40 non-repeated angry face trials, and thus took a total duration of 7 min 32 s.

### Image acquisition

Functional and structural MRI data were acquired using a 3T MRI system (Siemens, MAGNETOM Verio, Erlangen, Germany) equipped with a 16-channel head coil. Participants’ heads were cushioned with attached earmuffs. The functional images were obtained using a T2*-weighted gradient echo-planar imaging sequence (31 slices of 3.5-mm thickness and no gaps, repetition time [TR] = 2,000 ms, echo time [TE] = 30 ms, flip angle = 90°, image matrix = 124 × 124, field of view = 220 mm) with an in-plane resolution of 1.719 mm × 1.719 mm. Structural images with a resolution of 0.859 mm × 0.859 mm × 1.2 mm were acquired using a 3D T1-weighted gradient echo sequence (170 slices, TR = 9.692 ms, TE = 4.59 ms, image matrix = 224 × 224).

### Data analysis

#### Behavioral data

The behavioral data were analyzed according to the emotional valence of the face, the stimuli exposure, and the group. The three variables of interest were the emotional valence of the face (happy vs. angry), emotional status (repetition vs. transition), and group (SP vs. NC). The task performances, measured by accuracy and reaction time, were analyzed by a repeated measures analysis of variance (ANOVA) to assess the main effects of the three factors and their interactions using IBM SPSS Statistics for Windows, Version 20.0 (IBM SPSS Inc., Armonk, NY). Subsequent paired t-tests for post hoc analyses were performed to test the significance between the different conditions and groups.

#### Image data

Image preprocessing and statistical analysis were performed with Statistical Parametric Mapping software (SPM8; Wellcome Department of Cognitive Neurology, London, UK). After discarding the first six images from the dummy scan at each session, the remaining 220 images were used for further preprocessing. Differences in the slice acquisition time of the interleaved sequence were corrected, and realignment was performed to correct the artifact created by head motion. The corrected images were coregistered on the T1-weighted image of the same participant. The T1-weighted images were normalized to the standard T1 template, and the resulting transformation matrices were applied to the coregistered functional images. Functional data were smoothed with a Gaussian kernel of 8-mm full-width at half-maximum.

Preprocessed data were analyzed using a general linear model. Experimental trials were modeled separately using a canonical hemodynamic response function for individual data. Multiple linear regression, as implemented in SPM8 using a least-squares approach, was used to obtain the parameter estimates^80^. These estimates were then analyzed by testing specific contrasts using the participant as a random factor. According to the emotional valence and emotional status of the face, all trials were classified as HH, AH, AA, and HA trials. Images of the parameter estimates for each condition were created in the primary analysis, during which individual realignment parameters were entered as regressors to control for movement-related variance.

For the secondary analysis, the parameters for the four conditions, which were estimated in the primary analysis, and group condition were entered into the flexible factorial model, in which contrast maps were compared for group differences. The results were measured with group differences in relation to the emotional valence (happy and angry) and emotional status (repetition vs. transition) of the face. Significant results were determined by family-wise error (FEW) corrected p values of less than 0.001 and more than 100 voxels preferentially.

Post hoc tests for interactions and correlation analysis were performed on the a priori regions of interest (ROIs), which were defined as significant clusters within fronto-cingulate regions including the dACC [6, 10, 38] and DLPFC [48, 16, 32] related to cognitive control for emotional faces in group. The % BOLD signal changes in the ROIs were extracted in each condition using MarsBaR version 0.41 (http://marsbar.sourceforge.net), and the differences for the ROIs were analyzed using repeated measures ANOVA. The correlations between ROIs and behavioral error rate were calculated using Pearson correlation analyses in each condition and groups, and the p-values were adjusted by Benjamin–Hochberg FDR for multiple comparisons. Also, the statistical difference in regional correlation results between groups was computed after application of Fisher’s r-to-z transform.
